# Platelet Desialylation Is a Novel Mechanism and Therapeutic Target in *Daboia siamensis* and *Agkistrodon halys* Envenomation-Induced Thrombocytopenia

**DOI:** 10.3390/molecules27227779

**Published:** 2022-11-11

**Authors:** Cheng Zhang, Zhanfeng Zhang, Enyu Liang, Yunlong Gao, Hui Li, Fangfang Xu, Weiye Chen, Ming Liu, Xianzhang Huang

**Affiliations:** 1 Department of Laboratory Medicine, The Second Affiliated Hospital of Guangzhou University of Chinese Medicine, Guangzhou 510120, China; 2 Department of Laboratory Medicine, LKSKI-Keenan Research Centre for Biomedical Science, St. Michael’s Hospital, University of Toronto, Toronto, ON M5B 1W8, Canada; 3 Clinical Laboratory, The First Affiliated Hospital of Guangzhou University of Chinese Medicine, Guangzhou 510405, China; 4 Department of Hematology, The Second Affiliated Hospital of Guangzhou University of Chinese Medicine, Guangzhou 510120, China; 5 The Second Clinical School of Medicine Medicial College, Guangzhou University of Chinese Medicine, Guangzhou 510006, China; 6 School of Life Sciences, University of Science and Technology of China, Hefei 230027, China

**Keywords:** snake venom, platelet, macrophage, thrombocytopenia, desialylation, neuraminidase

## Abstract

Venom-induced thrombocytopenia (VIT) is one of the most important hemotoxic effects of a snakebite, which is often associated with venom-induced consumptive coagulopathy (VICC). Refractory thrombocytopenia without significant coagulation abnormalities has also been reported after envenomation by some viperid snakes; however, the mechanisms are not well understood and therapeutic strategies are lacking. Here, we found that patients injured by *Daboia siamensis* or *Agkistrodon halys* snakes, who were resistant to standard antivenom treatment, had developed coagulopathy-independent thrombocytopenia. Venoms from these viperid snakes, rather than from the elapid snake (*Bungarus multicinctus*), induced platelet surface expression of neuraminidase-1 (NEU-1), and significantly increased the desialylation of the glycoproteins on human platelets. The desialylated platelets caused by viperid snake venoms were further internalized by macrophages, which resulted in reduced platelet numbers in peripheral blood. Importantly, neuraminidase inhibitor significantly decreased viper venom-induced platelet desialylation, therefore inhibiting platelet phagocytosis by macrophages, and alleviating venom-induced thrombocytopenia. Collectively, these findings support an important role for desialylated platelet clearance in the progression of viper envenomation-induced, coagulopathy-independent thrombocytopenia. Our study demonstrates that the neuraminidase inhibitor may be a potential therapy or adjuvant therapy to treat snakebite-induced thrombocytopenia.

## 1. Introduction

Snakebites can cause various local and systemic diseases, leading to significant mortality and disability worldwide. For example, venoms from viperid snakes usually result in local tissue damage and systemic hemorrhage or cardiovascular shock, and venoms from elapid snakes mostly induce neuromuscular paralysis [1]. Venom-induced consumption coagulopathy (VICC) is the most common coagulopathy resulting from viper envenomation, which is considered to be induced by the activation of the coagulation pathway, mediated by toxins such as thrombin-like enzymes and prothrombin activators, and the consumption of clotting factors [2,3]. VICC and the related symptoms usually occur at the initial stage after snakebite and resolve rapidly following the administration of adequate doses of antivenom [4,5,6,7,8,9].

Tropical and subtropical areas have the highest snakebite incidence, especially in South and Southeast Asia [1,5,10,11]. *Daboia (D.) siamensis* and *Agkistrodon (A.) halys* belong to the viperid family. Snakebites from these snakes frequently occur in South and Southeast Asia [12]. Significant coagulation abnormalities, as well as low platelet counts (i.e., thrombocytopenia), have been frequently reported after a snakebite [13,14]. The initial onset of venom-induced thrombocytopenia (VIT) has often been thought to be associated with VICC, probably due to the fact that in vivo, blood coagulation and platelet activation are complementary and mutually dependent [15,16,17,18]. Previous studies also suggested that the primary etiology of VIT is likely the result of platelet aggregation/sequestration at the envenomation site [19]. Notably, however, several studies reported that VIT can occur and persist in the absence of significant coagulopathy after being bitten by rattlesnakes or five-pacer vipers [20,21], indicating that different mechanisms are involved.

In this study, we reported that patients injured by *D. siamensis* and *A. halys* snakes, who were resistant to standard antivenom treatment, had developed VIT that was not dependent on significant coagulation abnormalities. We further demonstrated that venoms from these viperid snakes markedly induced platelet desialylation, leading to their internalization by macrophages. Therefore, macrophage-mediated desialylated platelet clearance is critical for viper envenomation-induced, coagulopathy-independent thrombocytopenia, and neuraminidase inhibitors might be a potential therapy to alleviate the VIT.

## 2. Results

### 2.1. Daboia siamensis and Agkistrodon halys Envenomation Causes Persistent Thrombocytopenia without Inducing Significant Coagulopathy in Patients

**Figure 1:**
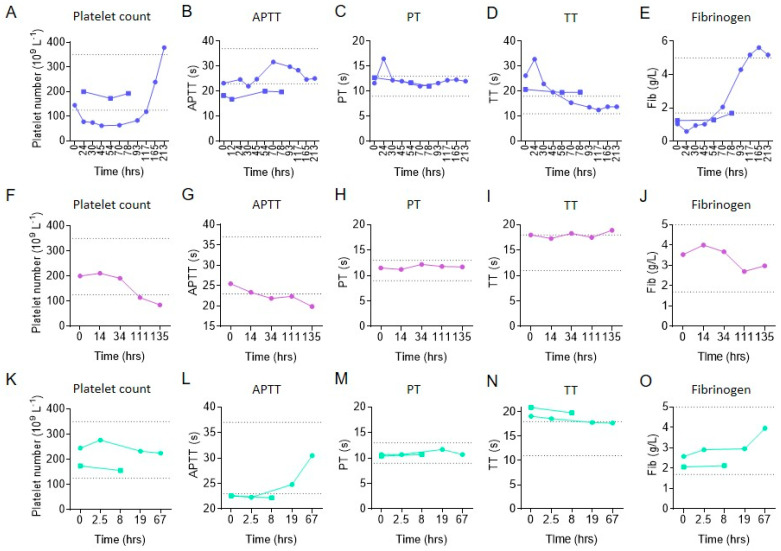
Effect of *Daboia siamensis*, *Agkistrodon halys* and *Bungarus multicinctus* envenomation on human peripheral platelet counts and blood coagulation. Peripheral platelet count (**A**,**F**,**K**), APTT (activated partial thromboplastin time, (**B**,**G**,**L**)), PT (prothrombin time, (**C**,**H**,**M**)), TT (thrombin time, (**D**,**I**,**N**)) and fibrinogen level (**E**,**J**,**O**) following a snakebite by *D. siamensis* (**A**–**E**), *A. halys* (**F**–**J**), *B. multicinctus* (**K**–**O**). The area between the dotted lines represents normal range. Each line represents one patient.

Thrombocytopenia refractory to the standard antivenom therapy has been reported after rattlesnake envenomation [20,22,23]. To investigate whether persistent thrombocytopenia occurs following bites by other snakes, we analyzed the clinical data of snakebite patients hospitalized in the First Affiliated Hospital of Guangzhou University of Chinese Medicine in 2020. Persistent thrombocytopenia was found in one of the two patients injured by *D. siamensis*, even after successive doses of antivenom treatment (Figure 1A, red line). Peripheral platelet numbers in this patient were maintained below the normal range until 117 h after hospitalization, while the blood coagulation was not significantly altered (Figure 1A–E). Another patient who was injured by *A. halys* developed a late, new-onset thrombocytopenia following regular antivenom therapy in which the platelet count dropped 111 h after hospitalization, while blood coagulation showed no obvious abnormality (Figure 1F–J). None of the two other patients injured by *B. multicinctus* had significantly decreased platelet counts or coagulopathy (Figure 1K–O). These data suggest that *D. siamensis* and *A. halys* caused persistent thrombocytopenia refractory to antivenom treatment, which, however, was independent of significant coagulopathy.

### 2.2. Macrophage Depletion Alleviates Viper Envenomation-Induced Thrombocytopenia In Vivo

**Figure 2:**
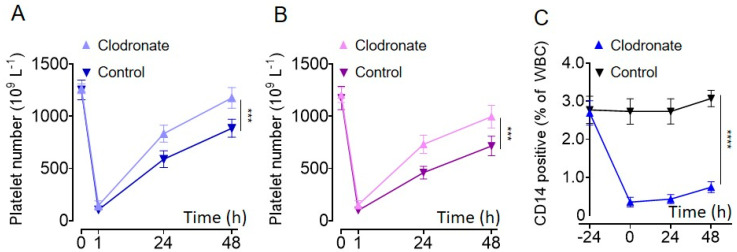
Macrophage depletion by clodronate alleviates viper envenomation-induced thrombocytopenia in vivo. Peripheral platelet count changes following the injection of venoms from (**A**) *D. siamensis* and (**B**) *A. halys* in mice that pre−treated with (dark blue or dark purple) or without (light blue or light purple) clodronate. (**C**) The percentage of CD14 macrophages following the treatment of liposomes that with or without clodronate. The results were presented as mean ± SD. Statistical analysis was performed with the area under the curve (AUC) using Student’s *t*-test. *n* = 6. *** *p* < 0.001, **** *p* < 0.0001.

The intraperitoneal injection of venoms from *D. siamensis* and *A. halys* led to severe thrombocytopenia in C57BL/6J mice (Figure 2). The average platelet numbers drastically dropped from 1253 to 104 × 10^9^/L at one hour after *D. siamensis* venom injection (Figure 2A), and from 1205 to 102 × 10^9^/L for *A. halys* venom (Figure 2B). Platelet counts gradually recovered; however, they were still approximately 40% less than the baseline, even 48 h after the injection (Figure 2).

It is known that macrophages or tissue-resident macrophages play important roles in platelet clearance [24,25,26]. To further investigate whether depleting macrophages could alleviate snake-venom-induced thrombocytopenia [27], we pre-injected mice with liposome-encapsulated clodronate. As expected, clodronate-containing liposomes, but not the liposome controls, dramatically decreased the CD14-positive macrophages within 24 h (Figure 2C). Importantly, clodronate pre-treatment significantly alleviated *D. siamensis* and *A. halys* venom-induced platelet clearance, suggesting that macrophages contribute to the platelet clearance caused by these venoms (Figure 2A,B).

### 2.3. Viper Venoms induce Platelet Desialylation and Platelet Internalization by Macrophages In Vitro

**Figure 3:**
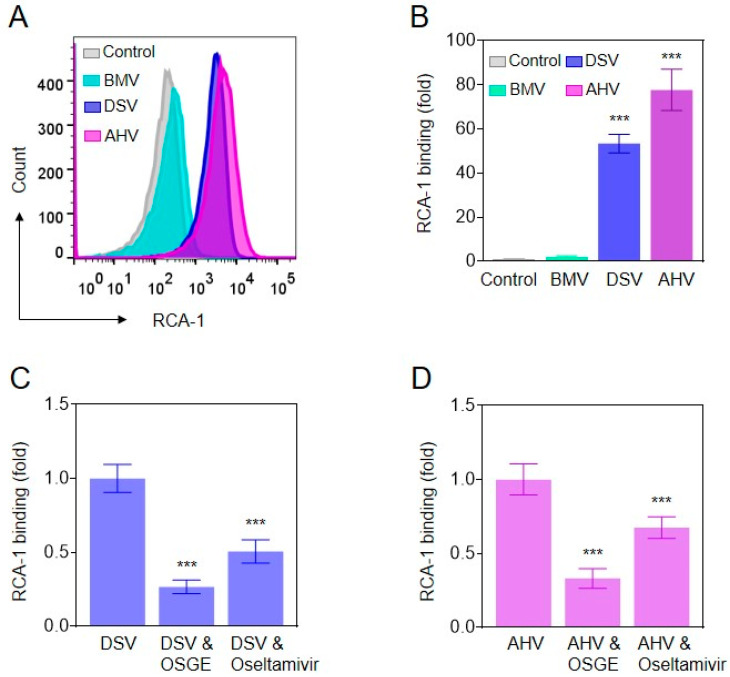
Viperid snake venoms induces platelet desialylation. (**A**,**B**) Effect of the venoms from *D. siamensis* (DSV), *A. halys* (AHV) and *B. multicinctus* (BMV) on RCA-1 binding. Effect of OSGE and oseltamivir on (**C**) DSV and (**D**) AHV venoms induced RCA-1 binding. Statistical analysis was performed with the one-way ANOVA test. *n* = 3. *** *p* < 0.001.

The desialylation of platelet membrane proteins is a mechanism of Fc-independent platelet clearance mainly involving macrophages [26,28,29]. To investigate whether venoms from *D. siamensis* and *A. halys* also induce platelet desialylation, we then tested the binding of the desialylation marker Ricinus communis agglutinin I (RCA-1) lectins [30,31,32] to platelets in the presence or absence of the venoms. We found that RCA-1 binding was profoundly increased by 53.35 and 77.67 fold on average by venoms from *D. siamensis* and *A. halys*, respectively, but not altered by *B. multicinctus* venom (Figure 3A,B). Interestingly, co-treatment by O-sialoglycoprotein endopeptidase (OSGE), which cleaves the glycosylated proteins on platelet membranes such as glycoprotein (GP) Ibα, GPVI, and α2β1 integrin, etc. [33,34], significantly reduced RCA-1 binding on venom-treated platelets (Figure 3C,D). These data suggest that desialylated O-glycan or O-glycan decorated proteins are responsible for snake venom-induced platelet desialylation. Furthermore, the sialidase (i.e., neuraminidase) inhibitor oseltamivir also markedly reduced RCA-1 binding, indicating that neuraminidase may mediate venom-induced platelet desialylation.

**Figure 4:**
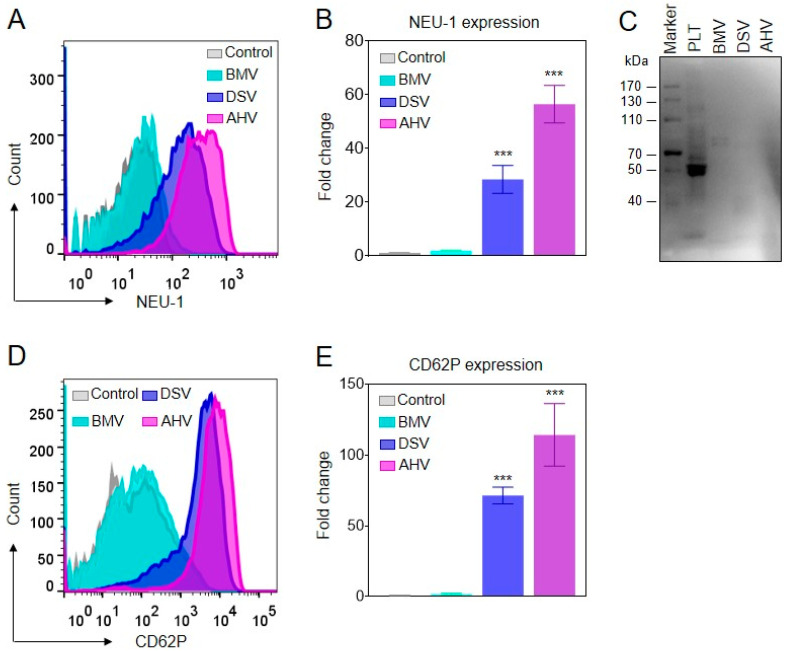
Effect of the Viperid snake venoms and elapid snake venom on platelet surface NEU-1 expression. (**A**,**B**) Effect of the venoms from *D. siamensis* (DSV), A. halys (AHV) and *B. multicinctus* (BMV) on platelet surface NEU-1 expression. (**C**) Expression of NEU-1 in platelets (PLT) and the snake venoms. (**D**,**E**) Effect of the snake venoms on platelet degranulation as reflected by CD62P (P-selectin) expression. Statistical analysis was performed with the one-way ANOVA test. *n* = 3. *** *p* < 0.001.

Notably, endogenous and exogenous neuraminidase regulates platelet desialylation under conditions such as immune thrombocytopenia and cold storage, etc. [31,35,36]. We, therefore, tested neuraminidase expression on platelet surfaces and snake venoms. Crude venoms from *D. siamensis* and *A. halys* rather than *B. multicinctus* significantly induced NEU-1 expression on human platelet surfaces (Figure 4A,B). The increased NEU-1 was not likely from the attached exogenous NEU-1, since neuraminidase was not detected by an NEU-1 polyclonal antibody in these venoms (Figure 4C). However, we also found that these viper venoms significantly induced platelet degranulation, as detected by platelet P-selectin (CD62P) expression (Figure 4D,E). Therefore, endogenous neuraminidase translocation from platelet granules to the membrane, induced by viper venoms, is the mechanism for platelet desialylation [31,37].

**Figure 5:**
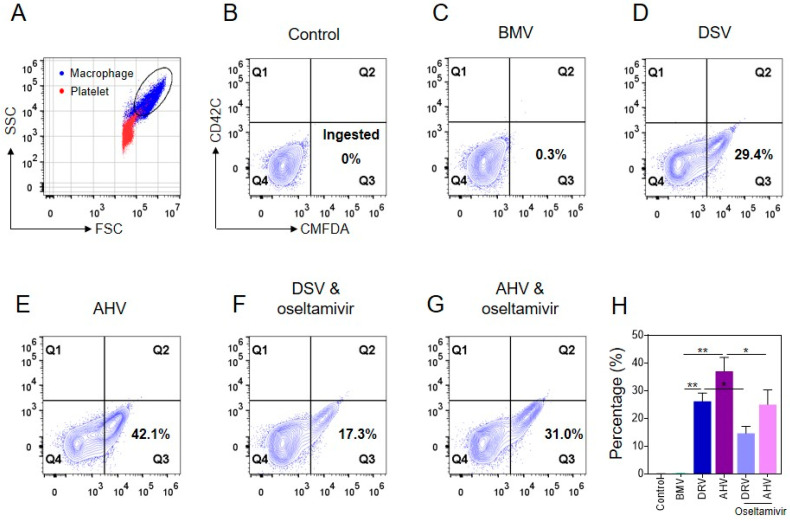
Effect of the Viperid snake venoms and elapid snake venom on platelet phagocytosis by macrophages. (**A**) Gating strategy of the platelets and macrophages. (**B**–**E**) Effect of the venoms from *D. siamensis*, *A. halys* and *B. multicinctus* on platelets phagocytosis by macrophages. (**F**,**G**) Effect of oseltamivir on *D. siamensis* and *A. halys* venom-induced platelet phagocytosis by macrophages. (**H**) Statistical analysis of the phagocytosis assays. Statistical analysis was performed with the one-way ANOVA test. *n* = 3. * *p* < 0.05, ** *p* < 0.01.

The tissue residential macrophage is critical in clearing desialylated platelets [28,38]. Therefore, we next examined whether venoms from *D. siamensis* and *A. halys* indeed induce platelet phagocytosis by macrophages. We discovered that crude venoms from the viperid snakes, rather than *B. multicinctus*, significantly induced platelet phagocytosis by RAW264.7 macrophages in vitro (Figure 5A–E). Importantly, the neuraminidase inhibitor oseltamivir significantly inhibited snake venom-induced platelet phagocytosis by macrophages (Figure 5F–H), suggesting that platelet desialylation induced by snake venoms is a critical mechanism contributing to macrophage-mediated platelet clearance, and that the clinically used neuraminidase inhibitor, oseltamivir, may alleviate snake venom-induced platelet clearance.

### 2.4. Macrophage Depletion Alleviates Viper Envenomation-Induced Thrombocytopenia In Vivo

**Figure 6:**
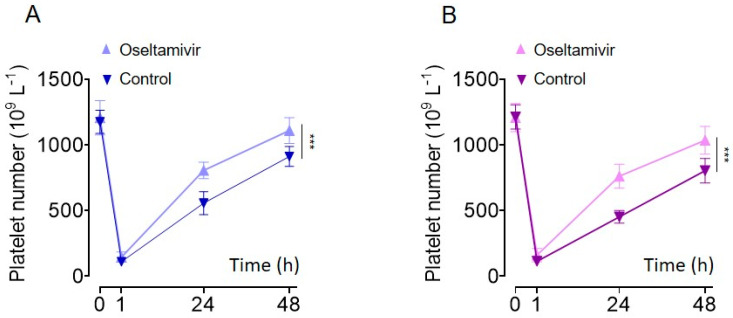
Oseltamivir phosphate significantly alleviates *D. siamensis* and *A. halys* venom−induced thrombocytopenia in mice. Peripheral platelet count changes following the injection of venoms from (**A**) *D. siamensis* and (**B**) *A. halys* with or without oseltamivir phosphate. The results were presented as mean ± SD. Statistical analysis was performed with the area under the curve (AUC) using the Student’s *t*-test. *n* = 6. *** *p* < 0.001.

Recent studies have demonstrated that oseltamivir acts on human sialidase (i.e., neuraminidase) which, therefore, alleviates refractory immune thrombocytopenia in humans by inhibiting platelet desialylation [39,40,41,42]. Since we observed that oseltamivir inhibited *D. siamensis* and *A. halys* venom-induced platelet phagocytosis by macrophages, we next investigated whether oseltamivir alleviated venom-induced thrombocytopenia. Consistently, oseltamivir significantly alleviated *D. siamensis* and *A. halys* venom-induced platelet clearance in a mouse model in vivo (Figure 6A,B), demonstrating that the neuraminidase-mediated platelet desialylation plays an important role in these snake venoms-induced thrombocytopenia. This result suggests that the sialidase inhibitor is a potential therapy to treat patients with snakebite-induced thrombocytopenia.

## 3. Discussion

*D. siamensis* and *A. halys* have been reported to cause VICC complicated by thrombocytopenia. Here, we found that persistent thrombocytopenia may occur independent of significant coagulopathy after a snakebite from *D. siamensis* and *A. halys*. Crude venoms from these snakes induced platelet desialylation, likely due to the increased neuraminidase expression on platelet surfaces. The desialylated platelets were internalized by macrophages, which could be alleviated by neuraminidase inhibitors.

Clinical features caused by *D. siamensis* and *A. halys* may range from local to systemic complications, including coagulopathy, thrombocytopenia, and bleeding [12,43,44]. Hemostatic disturbance is considered the most hemotoxic effect after the envenomation of these viperid snakes [3]. This is considered to be the result of coagulation factors and platelet consumption, induced by factor X activators, thrombin-like enzymes, and prothrombin activators in snake venoms. However, after antivenom treatment, the venom-induced consumption coagulopathy (VICC) usually resolves rapidly [3,4,5]. Clinical manifestations support that venom-induced thrombocytopenia (VIT) is usually associated with VICC. Interestingly, VIT may occur in the absence of significant coagulation abnormalities after rattlesnake envenomation [19,20,22,45]. It has also been documented that patients injured by some viperid snakes may develop late, new-onset thrombocytopenia [23,46,47]. Here, we demonstrated that macrophage-mediated desialylated platelet clearance is a novel mechanism and therapeutic target in *D. siamensis* and *A. halys* envenomation-induced thrombocytopenia.

Macrophages or tissue-resident macrophages play critical roles in platelet clearance in vivo. Macrophages not only phagocytose antibody-opsonized platelet via Fc receptors [24,25,26], but also internalize desialylated platelets through Ashwell-Morell receptors (AMR) and galactose lectin [28,31,35]. We found depletion of macrophages with liposome-encapsulated clodronate significantly shortened platelet recovery time (Figure 2), suggesting that macrophages are important for viper venom-induced thrombocytopenia. However, the depletion of macrophages did not significantly alter platelet numbers 1 h after venom injection (Figure 2), suggesting that macrophage-mediated platelet clearance is not significantly involved in the initial platelet sequestration after snakebite. The initial drop of platelet numbers may be due to platelet aggregation/sequestration caused by blood coagulation or platelet agonists in the venoms [19], while the persistent thrombocytopenia may be due to macrophage-related platelet clearance.

Platelet desialylation has recently been discovered as an Fc-independent mechanism that mediates platelet clearance by tissue resident macrophage [28,36,38]. Glycoproteins on platelets are decorated with sialic acid residues. Neuraminidases (i.e., sialidases) from either exogenous or endogenous origin can remove these sialic acid residues, therefore leading to platelet desialylation [40,48]. We found that venoms from *D. siamensis* and *A. halys*, rather than *B. multicinctus*, significantly induced platelet desialylation (Figure 3). Neuraminidase expression on platelet surfaces rather than in viper venoms was detected (Figure 4A–C); therefore, the desialylation of platelets may be caused by neuraminidase translocated from granules to platelet surfaces, as previously reported. In accordance with neuraminidase translocation, we found that these viper venoms induced significant platelet degranulation, as detected by P-selectin (CD62P) antibody (Figure 4D,E). Snake venoms are complex cocktails of bioactive molecules. Platelet-activating components, C-type lectin-like proteins or thrombin-like enzymes may induce or synergistically induce the translocation of endogenous neuraminidase from the inside to the surface, causing platelet desialylation [49].

Oseltamivir is an inhibitor of influenza viral neuraminidase, which has been used to treat influenza [50,51]. Recent studies revealed that oseltamivir also reduces human neuraminidase activation and alleviates desialylation-related immune thrombocytopenia [39,40,41,42]. We found that oseltamivir significantly inhibited *D. siamensis* and *A. halys* venom-induced platelet desialylation (Figure 3C,D), as well as macrophage-mediated platelet phagocytosis (Figure 5). Moreover, oseltamivir shortened platelet recovery time (Figure 6), suggesting that oseltamivir alleviates desialylation-related VIT. However, oseltamivir did not alter platelet numbers 1 h after the venom injection, suggesting that oseltamivir did not affect blood coagulation mediated platelet aggregation/sequestration. Therefore, the neuraminidase inhibitor may be a potential therapy or adjuvant therapy for snakebite-induced, and VICC-independent thrombocytopenia.

In conclusion, we found that the venoms from *D. siamensis* and *A. halys*, but not *B. multicinctus*, cause persistent/refractory thrombocytopenia in both humans and mice. The venoms trigger platelet desialylation by increasing the surface expression of neuraminidase (i.e., sialidase). Macrophage-mediated desialylated platelet clearance is, therefore, critical for viperid venom-induced persistent thrombocytopenia. Neuraminidase inhibitor decreased snake venom-induced platelet desialylation, and thus might be a promising strategy to alleviate viper envenomation-induced thrombocytopenia.

## 4. Materials and Methods

### 4.1. Snakebite Patients

The medical records of patients with snakebites from *D. siamensis*, *A. halys*, and *B. multicinctus* in the First Affiliated Hospital of Guangzhou University of Chinese Medicine, Guangdong (Southern China) in 2020 were retrospectively reviewed and evaluated. All the records were anonymized.

### 4.2. Material

Crude venoms from *D. siamensis*, *A. halys*, and *B. multicinctus* were obtained in Guangdong province, as we previously reported [52,53]. Liposome-encapsulated clodronate (LIP-CLOD) and liposome-encapsulated phosphate-buffered saline (LIP-PBS) were prepared, as previously described (40337ES08/10, YEASEN) [27].

### 4.3. Animal Studies

The animal protocol was approved by the Laboratory Animal Ethics Committee at the Second Affiliated Hospital of Guangzhou University of Chinese Medicine (2021018). The snake venoms were intraperitoneally injected into C57BL/6J mice (6–8 weeks old) at 0.5 mg/kg. Peripheral platelet counts were analyzed by Coulter Counter (Beckman Coulter). The injection of 0.1 mL/10 g (*v*/*w*) of LIP-CLOD through the tail-vein-induced complete depletion of splenic and hepatic macrophages in C57BL/6J mice within 24 h [27].

### 4.4. Platelet Desialylation Detection and NEU-1 Expression on Platelet Surface

Flow cytometry analysis of platelets was performed as we and others previously described [31,40,54]. The venoms from *D. siamensis*, *A. halys* and *B. multicinctus* were incubated with platelets with and without O-sialoglycoprotein endopeptidase (OSGE) or oseltamivir. Platelet desialylation level was detected by rhodamine-labeled ricinus communis agglutinin I (RCA-1) lectins (1/1000 dilution, RL-1082-5, VECTOR, Burlingame, CA, USA), which specifically binds to the galactose residues caused by glycoprotein desialylation [30,31]. NEU-1 expression on platelet surface was detected by NEU-1 antibody (OM210044, OmnimAbs, Alhambra, CA, USA) followed by secondary antibody conjugated with cy3 (711-165-152, Jackson, MS, USA).

### 4.5. Western Blot

The expression of NEU-1 in platelets lysate and snake venoms were determined by Western blot assay, as we previously described [51,55]. In brief, snake venoms (100 μg) or platelet lysate were denatured at 95 °C in loading buffer (50 mM Tris, 10% Glycerol, pH 6.8) with 1% β-mercaptoethanol and 2% SDS (*w*/*v*). Then, the denatured samples were loaded into 12% SDS-PAGE and immunoblotted with NEU-1 antibody (OM210044, OmnimAbs, Alhambra, CA, USA). NEU-1 was visualized by incubating with horseradish peroxidase (HRP)-conjugated secondary antibody (31460, Invitrogen, Waltham, MA, USA), followed by chemiluminescence (34579, Thermo, Waltham, MA, USA).

### 4.6. Platelet Internalization by Macrophages

Flow cytometry was used to analyze platelet phagocytosis by macrophages [26,56]. Briefly, Sepharose 2B chromatography column (CL2B300, Sigma, St. Louis, MO, USA) was used to generate gel-filtered platelets using mouse platelet-rich plasma (PRP) [57,58,59]. Platelets were stained with 5-chloromethylfluorescein diacetate (CMFDA) and incubated with RAW264.7 macrophages (ATCC, Manassas, VA, USA) for one hour at 37 °C in the presence or absence of the snake venoms. Then, RAW264.7 cells were washed by using Hanks’ balanced salt solution (HBSS), trypsinized (15050065, Gibco, Waltham, MA, USA), and stained with anti-CD42c antibody (X649, DyLight649, emfret). RAW264.7 with adherent platelets were CMFDA and DyLight649 double-positive, while RAW264.7 with completely phagocytized platelets was CMFDA-positive.

### 4.7. Statistical Analysis

GraphPad Prism (Version 8, La Jolla, CA, USA) was used for statistical Analysis. One-way ANOVA or Student’s *t*-test was used for statistical analysis. Mean ± standard deviation (SD) values were presented, and the significance was determined as * *p* <0.05, ** *p* < 0.01 or *** *p* < 0.001.

## Data Availability

Not applicable.

## Sample Availability

Samples of Oseltamivir, the antibodies and venoms used in this study are available from the authors.
